# The value of prebiopsy FDG-PET/CT in discriminating malignant from benign vertebral bone lesions in a predominantly oncologic population

**DOI:** 10.1007/s00256-020-03426-8

**Published:** 2020-04-06

**Authors:** Naila Loudini, Andor W. J. M. Glaudemans, Paul C. Jutte, Derya Yakar, Thomas C. Kwee

**Affiliations:** 1 Medical Imaging Center, Departments of Radiology, Nuclear Medicine and Molecular Imaging, University of Groningen, University Medical Center Groningen, |Hanzeplein 1, P.O. Box 30.001, 9700 RB Groningen, The Netherlands; 2 Department of Orthopedics, |University of Groningen, University Medical Center Groningen, Hanzeplein 1, P.O. Box 30.001, 9700 RB Groningen, The Netherlands

**Keywords:** 18F-FDG, Biopsy, Bone, PET-CT, Spine, Vertebra

## Abstract

**Purpose:**

To determine the value of prebiopsy ^18^F-fluoro-2-deoxy-D-glucose positron emission tomography (FDG-PET)/computed tomography (CT) in discriminating malignant from benign vertebral bone lesions.

**Materials and methods:**

This retrospective study included 53 patients with 55 vertebral bone lesions that underwent FDG-PET/CT before CT-guided biopsy. Pathologic examination of the biopsy sample and a minimum follow-up of 1 year were used as reference standard.

**Results:**

Sensitivity, specificity, positive predictive value, and negative predictive value of visual FDG-PET analysis (with lesion FDG uptake higher than liver FDG uptake as threshold for malignancy) in discriminating malignant from benign vertebral bone lesions were 91.3% (42/46), 22.2% (2/9), 85.7% (42/49), and 33.3% (2/6), respectively. The semiquantitative FDG-PET metrics SUV_max_ and SUV_peak_ achieved areas under the receiver operating characteristics curve of 0.630 and 0.671, respectively. Malignant lesions demonstrated bone lysis more frequently than benign lesions (60.9% (28/46) vs. 22.2% (2/9)), and this difference was nearly significant (*P* = 0.064). All other clinical and conventional imaging characteristics (including patient age, gender, previous diagnosis of malignancy, bone pain, weight loss, any CT abnormality, sclerosis, cortical destruction, bone marrow replacement, associated extraosseous soft tissue mass, and accompanying vertebral height loss, multiple bone lesions on FDG-PET/CT, and suspicious extraosseous lesions on FDG-PET/CT) were not significantly different (*P* = 0.143 to 1.000).

**Conclusion:**

FDG-PET/CT may steer the diagnosis (particularly thanks to a relatively high PPV and value of semiquantitative measurements), but cannot always classify vertebral bone lesions as malignant or benign with sufficient certainty. In these cases, biopsy and/or follow-up remain necessary to establish a final diagnosis.

## Introduction

Bone metastases are a frequent complication of cancer [1]. Importantly, the spine is the most common site of bone metastasis [2]. It has been reported that over 10% of patients with cancer develop a symptomatic spinal metastasis [2]. Meanwhile, there is also a wide spectrum of primary malignant tumors, benign tumors, and tumor-simulating lesions (e.g., infections and fractures) that can occur in the spine [3, 4].

If the combination of clinical and imaging characteristics is not specific for a clinically irrelevant benign spinal lesion such as a simple hemangioma, bone island, or Schmörl’s node, or another benign entity such as infection or fracture, percutaneous computed tomography (CT)-guided biopsy is often performed with the aim to establish a diagnosis. The diagnostic yield of CT-guided biopsy in the spine has been reported to range between approximately 60 and 80% [5–7] and to be lower than in other parts of the skeletal system [5, 6]. This is most likely due to the relatively smaller size of the vertebrae and to the difficulties in obtaining more than one biopsy core in this location [5]. Furthermore, overall complication rate of CT-guided biopsy in the spine is approximately 3% [8], with nerve root damage, local infection, pneumothorax, vascular injury, paraspinal hematoma, temporary paresis, paraplegia, meningitis, and even death among the spectrum of complications that may occur [7]. In addition, patients with spinal lesions may have pain and dyspnea and may not be able to lie still for the procedure to be successfully performed [9]. Although CT-guided biopsy remains indispensable in the diagnostic work-up of spinal lesions, its limitations and disadvantages indicate the need for ancillary noninvasive diagnostic methods for lesion characterization.

The noninvasive characterization of spinal lesions traditionally relies on structural patterns as seen on CT and magnetic resonance imaging (MRI) [3, 4]. Meanwhile, positron emission tomography (PET)/CT with the radiotracer ^18^F-fluoro-2-deoxy-D-glucose (FDG) is increasingly used in clinical practice [10]. FDG-PET provides information on lesion glucose metabolism, which goes beyond anatomical imaging [10]. However, there is a lack of data on its use for the evaluation of spinal lesions. It can be hypothesized that FDG uptake is higher in malignant than in benign spinal lesions, and that the need for vertebral biopsy in every patient can be reconsidered if FDG-PET/CT proves to achieve a high discriminatory performance in this setting.

The aim of this study was therefore to determine the diagnostic performance of prebiopsy FDG-PET/CT for the discrimination between malignant and benign vertebral bone lesions.

## Materials and methods

### Study design

This study was approved by the local institutional review board (IRB number 201800105), and because of its retrospective design, the requirement for informed consent was waived. The electronic patient database of our university hospital (University Medical Center Groningen, Groningen, the Netherlands) was searched for all patients who had an FDG-PET/CT scan within 2 months before CT-guided bone biopsy of a vertebral bone lesion, within a consecutive 8-year period (from August 2010 to September 2018). Lesions that were biopsied because of a priori high suspicion of spondylodiscitis, and lesions whose nature remained unclear even after biopsy and follow-up (see the “Reference standard” section), were excluded.

### FDG-PET/CT acquisition

All FDG-PET/CT scans were performed using an integrated PET/CT system (Biograph mCT 40- or 64-slice PET/CT, Siemens Healthineers, Erlangen, Germany). All patients fasted for at least 6 h and blood glucose levels were checked to be less than 11 mmol/L before 3 MBq FDG/kg body weight was intravenously administered. After approximately 60 min, low-dose unenhanced CT scanning (with 100 kV and an average tube current of 90 mAs) was performed for attenuation correction and anatomic correlation. Subsequently, 3D PET scanning was performed from mid-thigh to top of the skull using a multi-bed position approach (5 to 7 bed positions with 3 min per bed position). FDG-PET/CT acquisition and reconstruction were in line with the European Association of Nuclear Medicine/Research 4 Life guidelines [11].

### FDG-PET/CT evaluation

All FDG-PET/CT scans were interpreted by nuclear medicine physicians as part of standard clinical care. A research fellow (N.L.), supervised by a nuclear medicine physician (A.W.J.M.G), reviewed all FDG-PET/CT scans using a dedicated workstation with the Syngo.via software (Siemens Healthineers, Erlangen, Germany). This reader knew the location of each biopsied vertebral lesion, but was blinded to all other clinical, imaging, pathological, and follow-up findings. FDG uptake of all biopsied vertebral bone lesions was both visually and semiquantitatively assessed. Visual assessment was done using a four-point grading scale (1, no FDG uptake; 2, slight FDG uptake but less than mediastinal FDG accumulation; 3, FDG uptake higher than mediastinal FDG accumulation but lower than or equal to liver FDG uptake; 4, FDG uptake higher than liver FDG uptake). Quantitative assessment was done with (glucose corrected) maximum standardized uptake value (SUV_max_, representing the value of the single voxel with the highest SUV) and peak standardized uptake value (SUV_peak_, representing the mean SUV of a 12-mm diameter sphere encompassing the SUV_max_) measurements.

### CT-guided biopsy

Each vertebral bone lesion was biopsied under CT guidance by one of six musculoskeletal radiologists, as part of standard clinical care. Prebiopsy full-dose unenhanced CT (with 100–140 kV, and automatic dose modulation) was always performed to plan the biopsy route towards the lesion. One core biopsy was obtained from each lesion, with needle sizes ranging between 8 and 18 gauge, depending on the choice of the radiologist. Multislice CT systems (Somatom Sensation 16 or Definition 64 AS, Siemens Healthineers, Erlangen, Germany) and 8-gauge biopsy (Snarecoil, Ranfac, Avon, MA, USA), 11-gauge battery-powered biopsy (Arrow OnControl, Teleflex, Shavano Park, TX, USA), and 16- and 18-gauge biopsy (BioPince, Argon Medical Devices, Frisco, TX, USA) devices were used in this study.

### CT evaluation

Each vertebral bone lesion was reviewed on prebiopsy planning CT by a musculoskeletal radiologist (T.C.K.) using a dedicated workstation (Carestream Vue PACS, version 11.4.1.1102, Carestream Health, Rochester, NY, USA). This reader knew the location of each biopsied vertebral bone lesion, but was blinded to all other clinical, imaging, pathological, and follow-up findings. Reconstructed 2.0-mm axial, coronal, and sagittal CT images were reviewed in bone window (level, 700 HU; width, 3000 HU) and soft tissue (level, 40 HU; width, 500 HU) settings. The location of each lesion was first assessed for the presence of any CT abnormality. If this was the case, the lesion was then assessed for the presence or absence of lysis, sclerosis, cortical destruction, bone marrow replacement, associated extraosseous soft tissue mass, and accompanying vertebral height loss.

### Reference standard

The nature of each vertebral bone lesion was classified as malignant or benign based on the CT-guided biopsy sample, which was examined by a specialized musculoskeletal pathologist in each case. If the nature of the lesion could not be established with certainty based on the biopsy result, all available follow-up examinations (including subsequent tissue samplings, clinical and imaging tests) were used to determine whether the lesion of interest was malignant or benign. A lesion was considered malignant if a subsequent tissue sampling revealed malignancy. A lesion was considered benign if it remained stable or regressed with conservative treatment during a follow-up period of at least 1 year. The nature of all other vertebral bone lesions was considered unclear.

### Statistical analysis

Sensitivity, specificity, positive predictive value (PPV), and negative predictive value (NPV) of FDG-PET/CT in discriminating malignant from benign vertebral bone lesions were calculated, with a visual FDG uptake score of 4 (i.e., lesion FDG uptake higher than liver FDG uptake) considered to indicate malignancy and visual FDG uptake scores of 1 to 3 (i.e., lesion FDG uptake equal to or less than liver FDG uptake) considered to indicate benignancy. In addition, receiver operating characteristic curve analyses were done to calculate the areas under the curve (AUC) of the semiquantitative metrics SUV_max_ and SUV_peak_. Furthermore, the following variables were compared between malignant and benign vertebral bone lesions: patient age, patient gender, previous diagnosis of malignancy (before FDG-PET/CT and biopsy), reported bone pain, reported weight loss, previously described CT features, multiple bone lesions on FDG-PET/CT, and suspicious extraosseous lesions on FDG-PET/CT. These variables were analyzed using the Fisher exact test for binary data, the Mann-Whitney *U* test for nongaussian continuous data, and the unpaired *t* test for gaussian continuous data. Two-tailed *P* values less than 0.05 were considered statistically significant. Statistical analyses were executed using the MedCalc statistical software version 18.5 (MedCalc, Ostend, Belgium).

## Results

### Patients

Sixty-one patients underwent FDG-PET/CT within 60 days before CT-guided biopsy of a vertebral bone lesion. Of all the patients, only one experienced a complication: a pneumothorax that was due to needle malpositioning. None of the 61 patients was biopsied because of the suspicion of spondylodiscitis. Eight patients were excluded because the nature of the vertebral lesion remained unclear despite biopsy and follow-up (i.e., initial CT-guided biopsy results were indeterminate for a specific diagnosis, subsequent tissue samplings were not performed, and follow-up period was less than 1 year). Seven of these 8 patients either had a history of cancer (rectal cancer (*n* = 2), breast cancer (*n* = 1), breast cancer, neuroendocrine tumor and lymphoma (*n* = 1), and myeloma (*n* = 1)) and/or were suspected to have active cancer disease. Fifty-three patients (26 males and 27 females, with a median age of 61 years (age range, 3–79 years) at the time of biopsy) remained and were included in the analysis. Forty-five patients (84.9%) had a diagnosis of malignancy before FDG-PET and biopsy, as shown in Table 1. The indications for FDG-PET/CT were initial cancer staging (*n* = 20), general diagnostic problem solving (*n* = 9), cancer treatment response assessment (*n* = 8), suspicion of recurrent cancer (*n* = 8), restaging recurrent cancer (*n* = 7), primary tumor detection (*n* = 1), and surveillance/follow-up imaging after cancer treatment (*n* = 1). Note that one patient underwent FDG-PET/CT for initial cancer staging and because of suspicion of recurrent cancer at a later time point.

**Table 1:** Types of cancer in 45 patients of 53 included patients (84.9%) who had a diagnosis of malignancy before FDG-PET and biopsy

Types of cancer	Number of patients
Breast cancer	16
Lung cancer	7
Malignant lymphoma	5
Esophageal cancer	3
Head and neck cancer	3
Cholangiocarcinoma	1
Chordoma	1
Gastrointestinal stromal cell tumor	1
Melanoma	1
Multiple myeloma	1
Prostate cancer	1
Rectal cancer	1
Thymic neuroendocrine tumor	1
Urothelial carcinoma	1
Breast cancer and malignant lymphoma	1
Malignant lymphoma, melanoma, and mesothelioma	1

### Vertebral bone lesions

Of the 53 patients included, two underwent biopsy of two different vertebral bone lesions. For one of them, both biopsies were done in one session, while for the other, there was a time interval of 15 months between the two biopsy sessions. Therefore, the total amount of biopsied vertebral bone lesions was 55. Fifty lesions were located in the vertebral body, 2 lesions were located in the transverse spinous process, 2 lesions were located in the spinous process, and 1 lesion was located in the superior articular process. Eighteen lesions were at the thoracic level, 18 at the lumbar level, and 19 at the sacral level. Median number of days between FDG-PET/CT and biopsy was 12 days (range, 1–58 days). Forty-six vertebral lesions proved to be malignant (all diagnoses based on the CT-guided biopsy sample), and 9 lesions proved to be benign (all diagnoses based on a negative biopsy for malignancy and subsequent clinical and imaging follow-up). The nature of all vertebral bone lesions is shown in Table 2.

**Table 2:** Nature of 55 vertebral bone lesions

Nature of lesions	Number of lesions
Malignant (*n* = 46)	
Breast cancer	15
Lung cancer	12
Malignant lymphoma	3
Acute leukemia	2
Carcinoma of unknown primary	2
Esophageal cancer metastasis	2
Head and neck cancer metastasis	2
Myeloma/plasmacytoma	2
Chordoma	1
Ewing sarcoma	1
Melanoma metastasis	1
Neuroendocrine tumor metastasis	1
Renal cell cancer metastasis	1
Urothelial carcinoma metastasis	1
Benign (*n* = 9)	
Benign, but exact nature unknown	6
Osteoporotic fracture	2
Spondylodiscitis (with atypical presentation)Z	1

### Diagnostic value FDG-PET

**Fig. 1:**
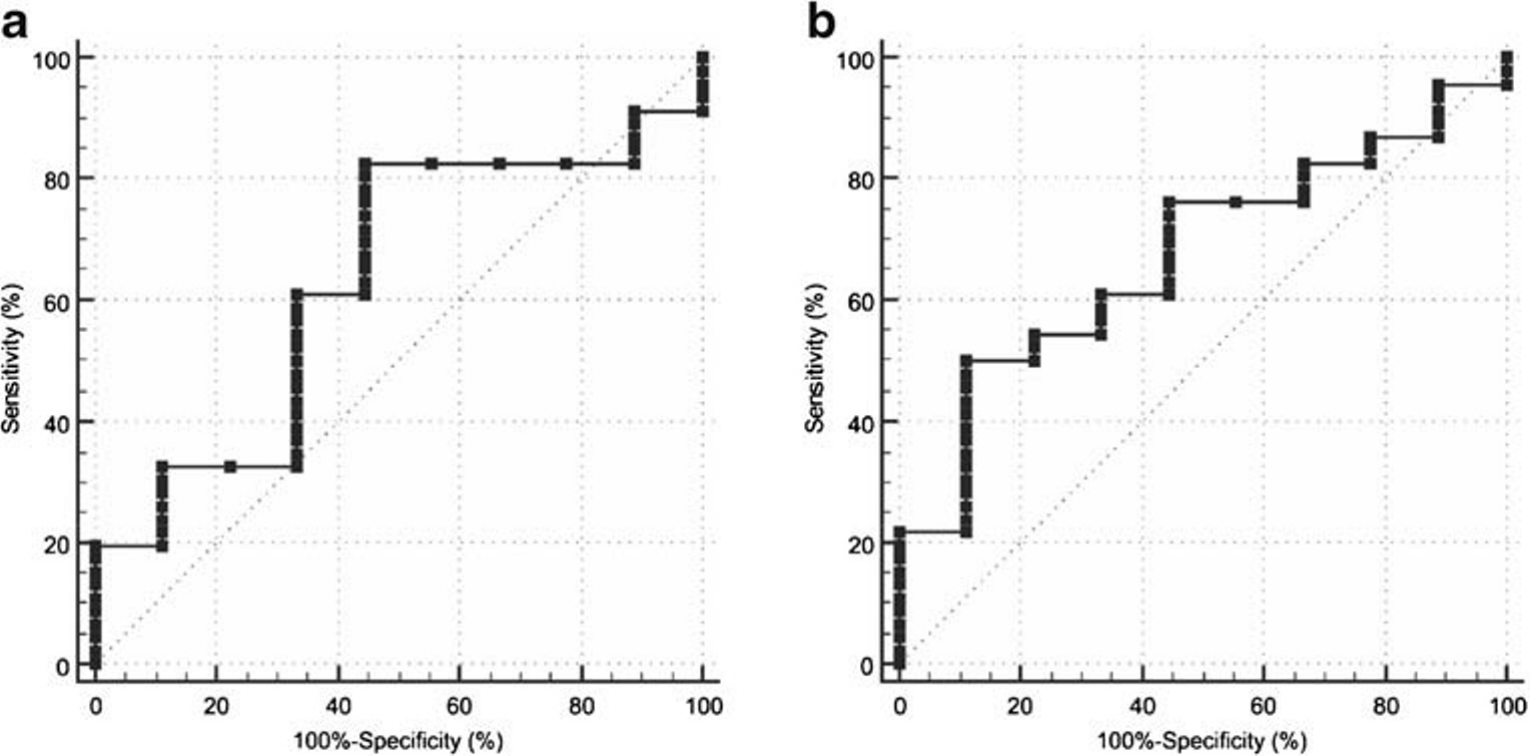
ROC curves of SUV_max_ (**a**) and SUV_peak_ (**b**) in discriminating malignant from benign vertebral bone lesions. AUCs were 0.630 (95% CI, 0.490–0.757) and 0.671 (95% CI, 0.532–0.792), respectively

**Fig. 2:**
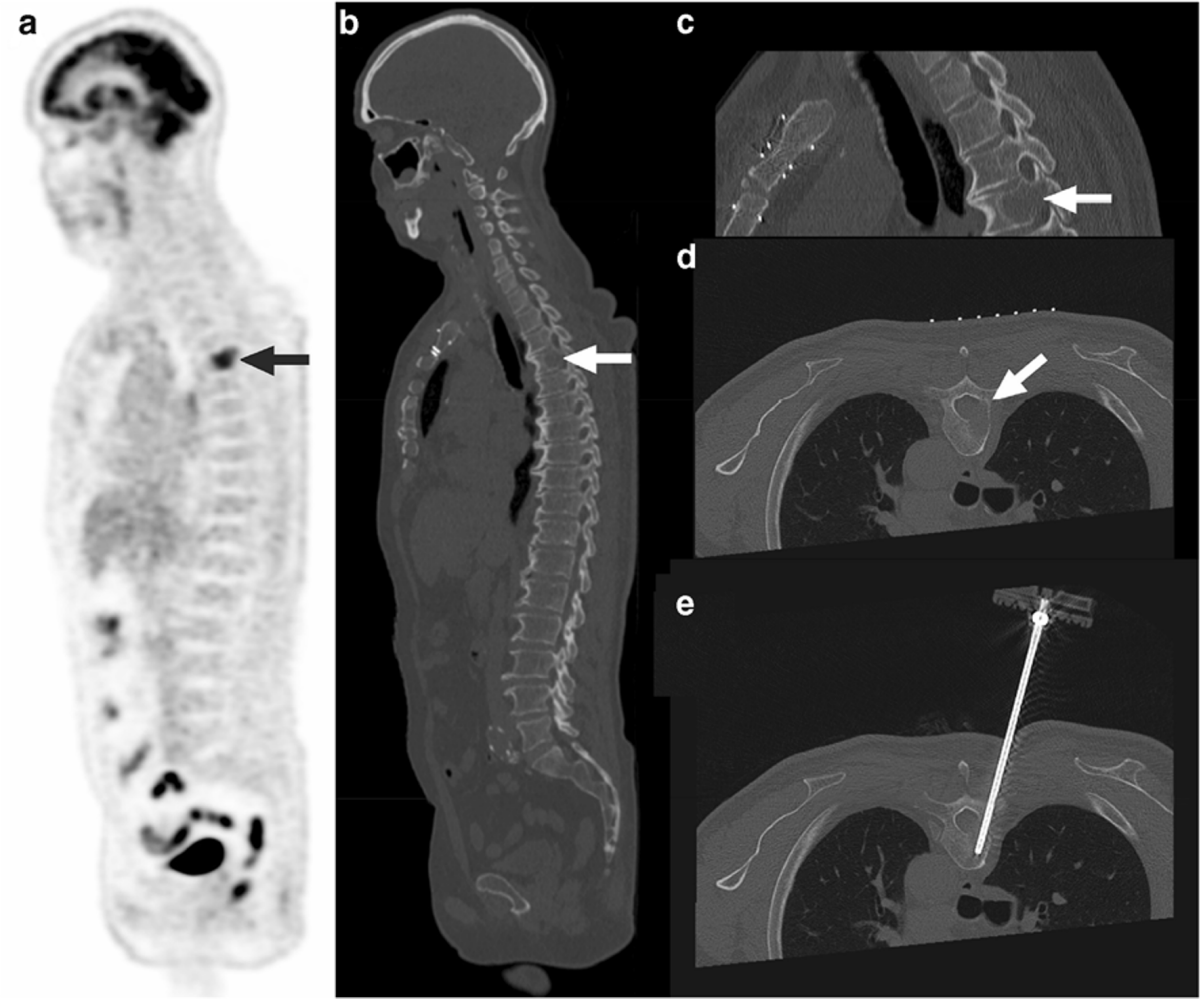
A 61-year-old man with a history of malignant lymphoma and melanoma, in whom FDG-PET showed a lesion in the T4 vertebra (**a**, arrow), also shown on low-dose CT (**b**, arrow). The lesion in the T4 vertebra had a visual FDG uptake higher than liver FDG uptake, an SUV_max_ of 11.1, and an SUV_peak_ of 9.4. Prebiopsy planning CT showed lysis, cortical destruction, and bone marrow replacement (**c** and **d**, arrows). The biopsy needle traverses the location of the lesion in vertebra T4 on CT (**f**). Pathologic examination revealed metastatic renal cell cancer

**Fig. 3:**
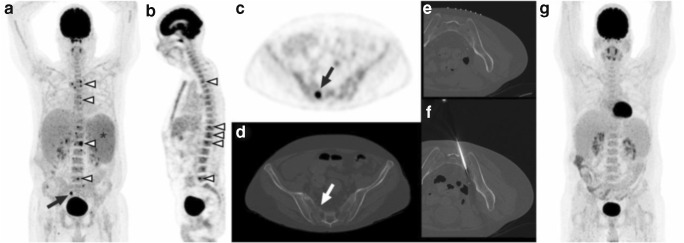
A 49-year-old man with a history of orthotopic liver transplantation because of alcoholic liver cirrhosis underwent FDG-PET because of fever of unknown origin and pancytopenia. FDG-PET showed multiple FDG-avid bone lesions, including involvement of several vertebrae (**a** and **b**, arrowheads). There is preexistent enlargement of the spleen, with diffusely increased splenic FDG uptake (**a**, asterisk). The lesion in vertebra S2 (**a** and **c**, arrows), with its anatomic location also shown on concomitantly acquired low-dose CT (**d**, arrow), was selected for CT-guided biopsy. This lesion had a visual FDG uptake higher than liver FDG uptake, an SUV_max_ of 16.5, and an SUV_peak_ of 8.0. Prebiopsy planning CT (**e**) showed no structural abnormality. The biopsy needle traverses the location of the lesion in vertebra S2 on CT (**f**). Pathologic examination neither showed any signs of malignancy or infection, nor could establish any other diagnosis. Follow-up FDG-PET 2 months later showed spontaneous resolution of all previously present FDG-avid bone lesions (**g**), hemoglobin, leukocyte, and thrombocyte levels had increased, and no malignancy or other active disease was diagnosed in the next 36 months. Therefore, although the nature of the abnormality in vertebra S2 remained unclear (with hyperplastic red bone marrow islands in the differential diagnosis), it could be classified as benign

**Fig. 4:**
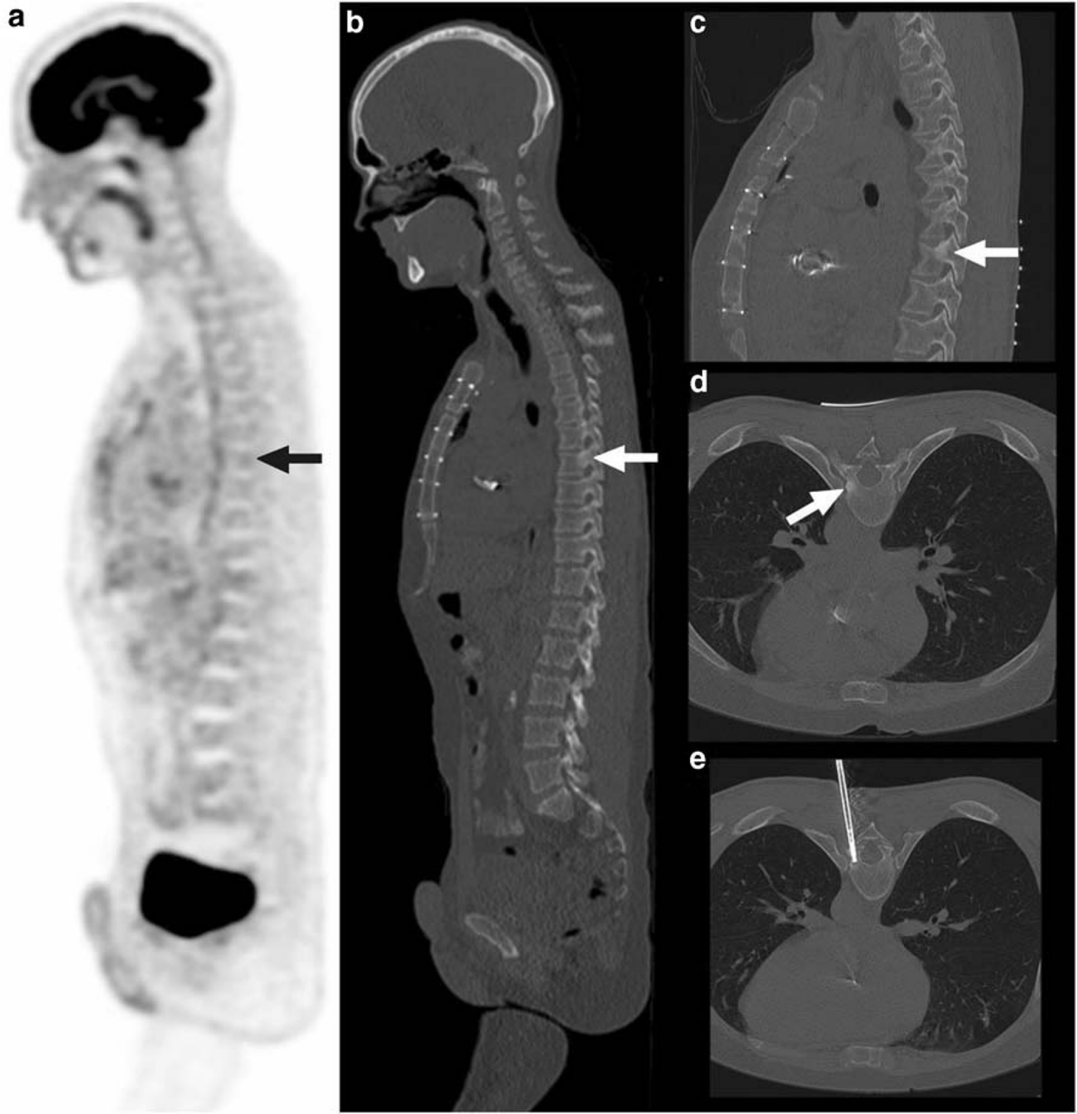
A 41-year-old man with multiple endocrine neoplasia type 1 underwent FDG-PET to monitor a known neuroendocrine tumor in the pancreas and previously seen FDG-avid mediastinal lymph nodes. These findings were stable on FDG-PET, but on the low-dose CT part of the FDG-PET examination, a sclerotic lesion in the left dorsal T8 vertebra—left pedicle—was seen, without any visual FDG uptake (**a** and **c**, arrows). Corresponding SUV_max_ was 2.1 and SUV_peak_ was 1.1. The lesion is more clearly visualized on prebiopsy planning CT (**c** and **d**, arrows). The biopsy needle is shown to traverse the location of the lesion in vertebra T8 on CT (**e**). Pathologic examination revealed metastatic neuroendocrine tumor

Sensitivity, specificity, PPV, and NPV of visual FDG-PET/CT analysis in discriminating malignant from benign vertebral bone lesions were 91.3% (42/46), 22.2% (2/9), 85.7% (42/49), and 33.3% (2/6), respectively. The semiquantitative FDG-PET metrics SUV_max_ and SUV_peak_ achieved AUCs of 0.630 (95% confidence interval (CI), 0.490–0.757) and 0.671 (95% CI, 0.532–0.792), respectively. Corresponding ROC curves are shown in Fig. 1. Representative case examples are shown in Figs. 2, 3, and 4.

### Diagnostic value of clinical and conventional imaging characteristics

Bone lysis was more frequently observed in malignant vertebral bone lesions (60.9% (28/46)) than in benign vertebral bone lesions (22.2% (2/9)), and this difference was nearly significant (*P* = 0.064). All other clinical and conventional imaging characteristics were not significantly different between malignant and benign vertebral bone lesions (*P* = 0.143 to 1.000), as shown in Table 3.

**Table 3:** Comparison of patient and conventional imaging characteristics between malignant and benign vertebral bone lesions

Characteristic*	Malignant	Benign	*P* value
Age (years), median	61^a^	65^a^	0.265^b^
Male sex	23/46 (50.0%)	5/9 (55.5%)	1.000^c^
Previous diagnosis of malignancy	38/46 (82.6%)	7/9 (77.7%)	0.661^c^
Bone pain	19/46 (41.3%)	4/9 (44.4%)	1.000^c^
Weight loss	5/46 (10.9%)	2/9 (22.2%)	0.321^c^
CT abnormality (any)	37/46 (80.4%)	6/9 (66.7%)	0.392^c^
CT lysis	28/46 (60.9%)	2/9 (22.2%)	0.064^c^
CT sclerosis	19/46 (41.3%)	5/9 (55.5%)	0.482^c^
CT cortical destruction	29/46 (63.0%)	3/9 (33.3%)	0.143^c^
CT bone marrow replacement	17/46 (37.0%)	2/9 (22.2%)	0.473^c^
CT associated soft tissue mass	10/46 (21.7%)	2/9 (22.2%)	1.000^c^
CT accompanying vertebral height loss	8/46 (17.4%)	2/9 (22.2%)	0.661^c^
Multiple bone lesions on FDG-PET/CT	26/46 (56.5%)	4/9 (44.4%)	0.716^c^
Suspicious extraosseous lesions on FDG-PET/CT	33/46 (71.7%)	5/9 (55.5%)	0.435^c^
Glucose corrected SUV_max_, median	10.0^a^	6.0^a^	0.219^b^
Glucose corrected SUV_peak_, median	8.1^a^	4.4^a^	0.106^b^

^*^Patient and imaging characteristics corresponding to each of 55 separately biopsied vertebral bone lesions

^a^Not normally distributed according to Shapiro-Wilk test

^b^Mann-Whitney *U* test

^c^Fisher exact test

## Discussion

This study aimed to elucidate the role of prebiopsy FDG-PET/CT in characterizing vertebral bone lesions. Importantly, the fact that FDG-PET/CT was performed before biopsy in all patients in this study implies that the observation of an FDG-avid spinal lesion frequently prompted the clinical decision to perform a vertebral biopsy. Patients with FDG-negative or low FDG-avid vertebral bone lesions (e.g., simple hemangioma, bone island, or Schmörl’s node) that have characteristic conventional imaging characteristics are usually not selected to undergo vertebral biopsy and were hence not included in the present study. This explains that 49 out of 55 vertebral bone lesions in this study had the highest visual FDG uptake score (i.e., lesion FDG uptake exceeding liver FDG uptake). However, this represents clinical practice, and the included patients are those in whom improved noninvasive lesion characterization is paramount. Since this results in a natural overrepresentation of FDG-avid lesions compared with FDG-negative or low FDG-avid lesions among the total amount of biopsied vertebral bone lesions, PPV is the most important diagnostic metric in this setting.

The results of our study show that FDG-PET/CT achieves a fairly high, but not infallible PPV of 85.7% (95% CI, 80.7–89.6%) in discriminating malignant from benign vertebral bone lesions. This is in line with previous work that has shown that there are several benign bone conditions that may be FDG-avid and mimic malignancy, including (focal) red marrow hyperplasia [12]. Semiquantitative FDG-PET/CT measurements had some value in differentiating malignant from benign vertebral bone lesions, with AUCs of 0.630 and 0.671 for SUV_max_ and SUV_peak_, respectively. Clinical and other imaging findings did not prove to be diagnostically useful, except that a lytic appearance tended to more frequently occur in malignant than in benign vertebral bone lesions (60.9% vs. 22.2%, respectively). Overall, FDG-PET/CT certainly has value in characterizing vertebral bone lesions. However, for patients whose treatment decisions depend on the nature of an FDG-avid vertebral lesion (as detected on PET/CT), and SUV values are not in the very high range, it is still advocated to perform a biopsy to confirm malignancy and to rule out a benign cause. On the other hand, biopsy does not always provide a definitive diagnosis. In the case of benign vertebral lesions for instance, it is not infrequently difficult to establish a specific diagnosis based on a limited amount of tissue obtained. Follow-up is then necessary to confirm benignancy, as demonstrated in the present study. This indicates the need for improved diagnostic tools, such as PET/MRI, artificial intelligence-based methods, and perhaps tumor-specific tracers, which should be the topics of further research.

Some previous studies have investigated the value of FDG-PET/CT for the characterization of bone lesions, with CT-guided biopsy as reference standard [13–16]. In one of the largest of these studies, Adams et al. [14] analyzed 94 patients with bone lesions that exceeded liver FDG uptake. Bone biopsy showed a malignant lesion in 83 patients, which corresponded to a PPV for malignancy of 88.3% (95% CI, 80.1–93.5%) [14], which approaches the PPV found in the present study. However, the study by Adams et al. [14] and most other previous studies on this topic included patients with bone lesions in all parts of the skeleton without a separate analysis for vertebral lesions [13, 15, 16]. Since the spectrum and incidence of diseases in the spine are different than in other bones [3, 4], a separate analysis for vertebral bone lesions is required. Another study by Laufer et al. [17] evaluated the accuracy of FDG-PET in the diagnosis of vertebral metastases in 82 patients with previously diagnosed cancer, with CT-guided biopsy as reference standard [17]. FDG-PET was reported to have a PPV of 97.4% (95% CI, 93.4–99.0%) for malignancy when considering all lesions with an SUV of 2.0 as malignant [17]. However, this very high PPV is subject to inflation due to the fact that the SUV_max_ threshold of 2.0 was retrospectively applied to the dataset. In addition, SUV_max_ measurements were done by different readers (in part obtained from the clinical radiology reports); it was not reported if these measurements were performed in a blinded manner; an outdated non non-cross-calibrated stand-alone PET system was used, and reconstructed images were not performed according to EANM/EARL standards, which all shed doubts on the validity of their results.

The present study had several limitations. First, despite the 8-year inclusion period, the number of patients with benign vertebral bone lesions was relatively low. This is due to the general low incidence of benignancy in vertebral lesions that are selected for biopsy, and the fact that several patients had to be excluded because the nature of their vertebral lesions remained unclear despite biopsy and follow-up. The majority of patients (84.9%) had a medical history of cancer, which limits the extent to which the conclusion of the study can be applied to the general population. Nevertheless, to the best of our knowledge, it is still the largest study on this topic so far. Second, the results of this study are not applicable to the differentiation between benign and malignant vertebral compression fractures, because only 10 of 55 included vertebral lesions had accompanying vertebral height loss (of which only 4 with severe, more than 40%, height loss [18]), which did not allow for a subanalysis. Third, this study was performed at a tertiary care center in Europe, and the results may be different in other institutions with different patient populations and with different types of vertebral pathology. Fourth, the additional value of MRI to FDG-PET (and vice versa) could not be assessed, because only 19 of 55 lesions were imaged on MRI. Fifth, no comparison was made with planar or SPECT/CT bone scintigraphy. However, bone scintigraphy reflects indirect evidence for disease as a result of reactive bone formation after long-standing red marrow involvement [19, 20]. In contrast, FDG-PET detects metastasis very early on during the course of disease when it is confined to the bone marrow [19, 20]. In addition, unlike bone scintigraphy, FDG-PET allows for assessment of both skeletal and extraskeletal lesions [19, 20]. Therefore, bone scintigraphy has important limitations. In our study population, only 4 patients have also undergone bone scintigraphy before FDG-PET/CT.

In conclusion, FDG-PET/CT may steer the diagnosis (particularly thanks to a relatively high PPV and value of semiquantitative measurements), but cannot always classify vertebral bone lesions as malignant or benign with sufficient certainty. In these cases, biopsy and/or follow-up remain necessary to establish a final diagnosis.
